# Thinking beyond cut-off scores in the assessment of potentially addictive behaviors: A brief illustration in the context of binge-watching

**DOI:** 10.1556/2006.2023.00032

**Published:** 2023-06-30

**Authors:** Pauline Billaux, Joël Billieux, Stéphanie Baggio, Pierre Maurage, Maèva Flayelle

**Affiliations:** 1 Louvain Experimental Psychopathology Research Group (LEP), Psychological Science Research Institute, UCLouvain, Louvain-la-Neuve, Belgium; 2 Institute of Psychology, University of Lausanne, Lausanne, Switzerland; 3 Institute of Primary Health Care (BIHAM), University of Bern, Bern, Switzerland; 4 Population Health Laboratory (#PopHealthLab), University of Fribourg, Fribourg, Switzerland

**Keywords:** behavioral addictions, addictive behaviors, binge-watching, cut-off scores

## Abstract

While applying a diagnostic approach (i.e., comparing “clinical” cases with “healthy” controls) is part of our methodological habits as researchers and clinicians, this approach has been particularly criticized in the behavioral addictions research field, in which a lot of studies are conducted on “emerging” conditions. Here we exemplify the pitfalls of using a cut-off-based approach in the context of binge-watching (i.e., watching multiple episodes of series back-to-back) by demonstrating that no reliable cut-off scores could be determined with a widely used assessment instrument measuring binge-watching.

The recent expansion of the behavioral addiction research field is a concern (Billieux, Flayelle, & King, 2022). Binge-watching (i.e., watching multiple episodes of series in one session) research exemplifies this phenomenon through the development of various assessment tools reclaiming traditional substance-use disorder criteria. This trend led to the conceptualization of binge-watching as a potential addictive behavior (e.g., Forte, Favieri, Tedeschi, & Casagrande, 2021; Orosz, Bőthe, & Tóth-Király, 2016; Paschke, Napp, & Thomasius, 2022; Starosta, Izydorczyk, & Lizińczyk, 2019). Other studies, however, insisted on the need to distinguish elevated (but non-harmful) binge-watching from problematic binge-watching in order to prevent over-pathologization (Flayelle et al., 2022; Steins-Loeber, Reiter, Averbeck, Harbarth, & Brand, 2020; Töth-Király, Böthe, Töth-Fáber, Gÿozö, & Orosz, 2017). The Binge-Watching Engagement and Symptoms Questionnaire (BWESQ; Flayelle et al., 2019) is a quantitative tool that assesses this dual nature of binge-watching (i.e., healthy vs. problematic) by measuring both healthy engagement (e.g., positive emotions, pleasure preservation) and symptoms of problematic binge-watching (e.g., loss of control, dependency). As the BWESQ is increasingly used in various contexts (e.g., Alfonsi et al., 2022; Boursier et al., 2021; Costa, Bugatti, & Lucchini, 2022; Demir & Batik, 2020; Gabbiadini, Baldissarri, Valtorta, Durante, & Mari, 2021; Munawar & Siraj, 2022; Tolba & Zoghaib, 2022), dozens of researchers have recently requested cut-off scores to identify problematic binge-watching. However, although following a diagnostic approach (i.e., comparing “clinical” cases with “healthy” controls) is core to psychiatry research and clinical practice, such an approach has been criticized in relation to putative behavioral addictions (Billieux, Schimmenti, Khazaal, Maurage, & Heeren, 2015), especially because these behaviors concern daily life activities and leisure, which can be performed at high levels of engagement without involving negative consequences and functional impairment (Bőthe, T.th-Kir.ly, Orosz, Potenza, & Demetrovics, 2020; Brevers, Maurage, Kohut, Perales, & Billieux, 2022; Charlton & Danforth, 2007; Whelan, Laato, Islam, & Billieux, 2021). Although the BWESQ was not developed as a diagnostic tool, we addressed this request by exploring whether reliable BWESQ cut-off scores could be determined.

We capitalized on an international data set comprising 12,616 BWESQ answers from series viewers (Flayelle, Castro-Calvo, et al., 2020). We applied the criteria from prior work on binge-watching (Billaux, Billieux, Gärtner, Maurage, & Flayelle, 2022; Flayelle, Verbruggen, et al., 2020)^1^ to distinguish three groups: 1) *non-binge-watchers* (*n* = 2,642), with a typical viewing session comprising less than three episodes and lasting for less than 2 h, with neither a reported functional impact caused by series watching nor self-identification as problematic series viewers; 2) *trouble-free binge-watchers* (*n* = 2,345), with a typical viewing session comprising three or more episodes and lasting at least 2 h per viewing session without reporting a functional impact caused by series watching and without self-identifying as problematic series viewers; and 3) *problematic binge-watchers* (*n* = 2,996), with a typical viewing session comprising three or more episodes and lasting at least 2 h, with a reported functional impact caused by series watching. This classification approach resulted in a final sample size of 7,983 participants (Age_M(SD)_ = 24.19 (7.91), 70.90% female). We thus excluded the remaining 4,633 participants who did not fulfill the criteria related to any of the three groups (e.g., participants who typically watched less than two episodes but for more than 2 h). However, because cut-off scores aim at dissociating clinical from non-clinical populations, we gathered *non-binge-watchers* and *trouble-free binge-watchers* into one group of *non-problematic TV series viewers* (*n* = 4,987, Age_M(SD)_ = 24.74 (8.49), 67.70% female), in opposition to the group of *problematic binge-watchers* (*n* = 2,996, Age_M(SD)_ = 23.28 (6.74), 76.30% female).

We conducted accuracy analyses for each of the seven BWESQ facets: *binge-watching* (e.g., “I always need to watch more episodes to feel satisfied”), *dependency* (e.g., “I am usually in a bad mood, sad, depressed or annoyed when I can't watch any TV series, and I feel better when I am able to watch them again”), *desire/savoring* (e.g., “I get really excited when a new episode is released”), *engagement* (e.g., “In my opinion, TV series are a part of my life and they contribute to my welfare”), *loss of control* (e.g., “I watch more TV series than I should”), *pleasure preservation* (e.g., “I worry about getting spoiled”), and *positive emotions* (e.g., “Watching TV series is a cause for joy and enthusiasm in my life”).

Using SPSS 27.0 (IBM, Corp.), we first assessed the diagnostic accuracy with area under the curve (AUC) analyses of receiver operating characteristics (ROC) curves, following diagnostic accuracy guidelines (i.e., AUC <0.70 implying low accuracy, AUC ≥0.70 and <0.90 indicating moderate diagnostic accuracy, and AUC ≥0.90 corresponding to high diagnostic accuracy; Swets, 2014). Results indicated low or close to low accuracy for the following five facets: *engagement* (AUC = 0.70), *dependency* (AUC = 0.68), *desire/savoring* (AUC = 0.72), *positive emotions* (AUC = 0.66) *and pleasure preservation* (AUC = 0.62). Because *loss of control* (AUC = 0.82) and *binge-watching* (AUC = 0.81) had moderate diagnostic accuracy, we conducted further accuracy analyses: specificity, sensitivity, positive predictive value (PPV), and negative predictive value (NPV). As observed in Figs 1 and 2, and based on accuracy indices for each of the curve coordinates (see Appendixes A and B), a cut-off score of 15.50 (corresponding to an actual score of 16) optimizes the accuracy of both subscales, ensuring a minimization of false positives (contrarily to the values inferior to the 15.50 cut-off score). For the *loss of control* facet, this threshold yields a poor sensitivity score of 54.40% (yielding a rate of 45.60% false negatives), a more than acceptable specificity score of 89.30%, a medium PPV of 75.30%, and a medium NPV of 76.50%. Regarding the *binge-watching* facet, this threshold is related to poor sensitivity (56.10%, yielding 43.90% false negatives), a good specificity score (86.30%), and a medium PPV (71.20%) and NPV (76.60%). This implies that if clinicians were to use either the *binge-watching* or *loss of control* subscale for screening purposes, approximately 30% of respondents labeled as presenting problematic binge-watching would be misclassified (Maraz, Király, & Demetrovics, 2015). Considering such a substantial likelihood of generating false positives, we therefore cannot reasonably recommend the use of cut-off values for the *binge-watching* and *loss of control* facets of the BWESQ.

**Fig. 1.**
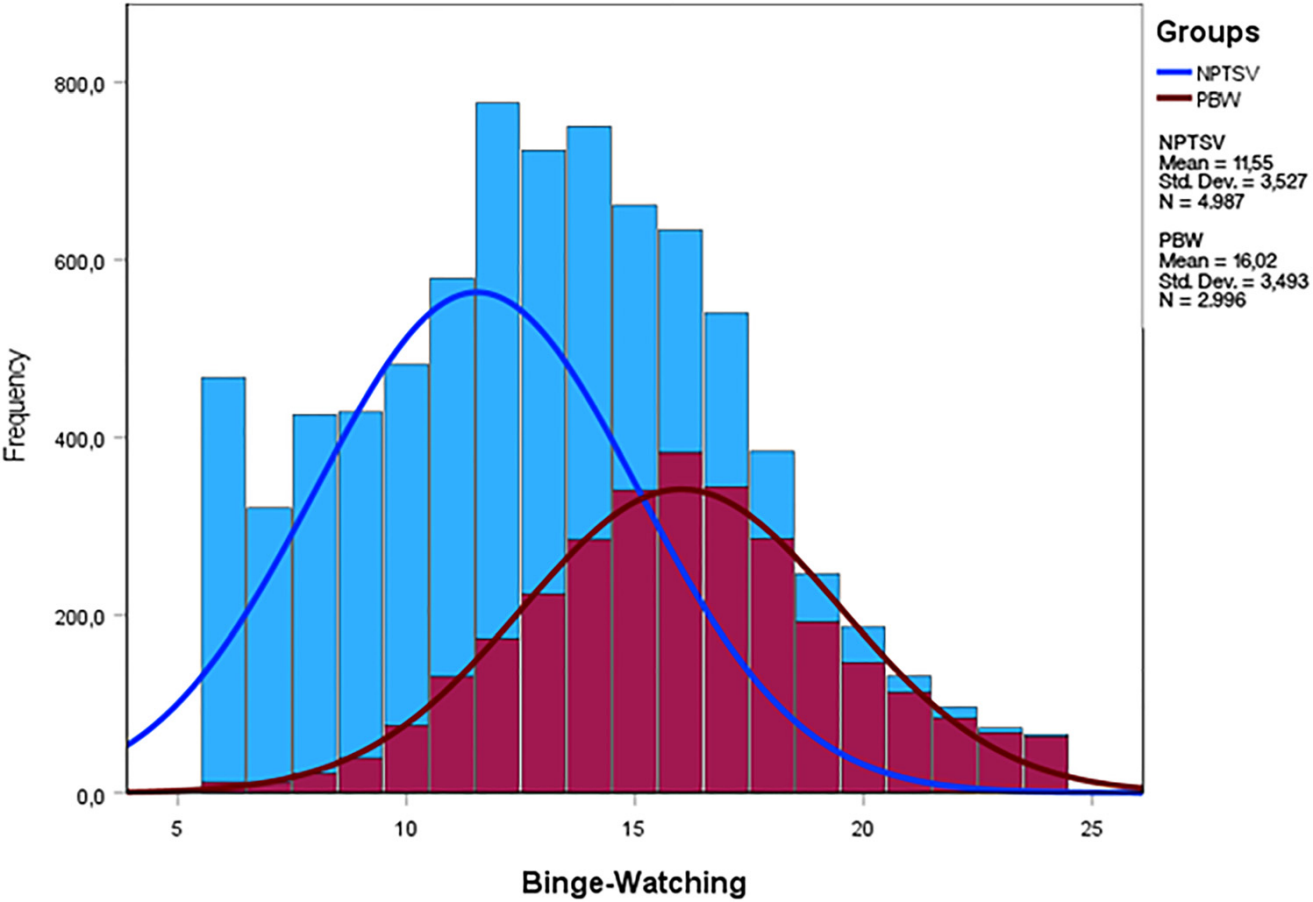
Frequency curves of scores of non-problematic TV series viewers and problematic binge-watchers for the binge-watching facet of the BWESQ *Note*. BWESQ: Binge-Watching Engagement and Symptoms Questionnaire; PBW: problematic binge-watchers; NPTSV: non-problematic TV series viewers; Std. Dev.: standard deviation

**Fig. 2.**
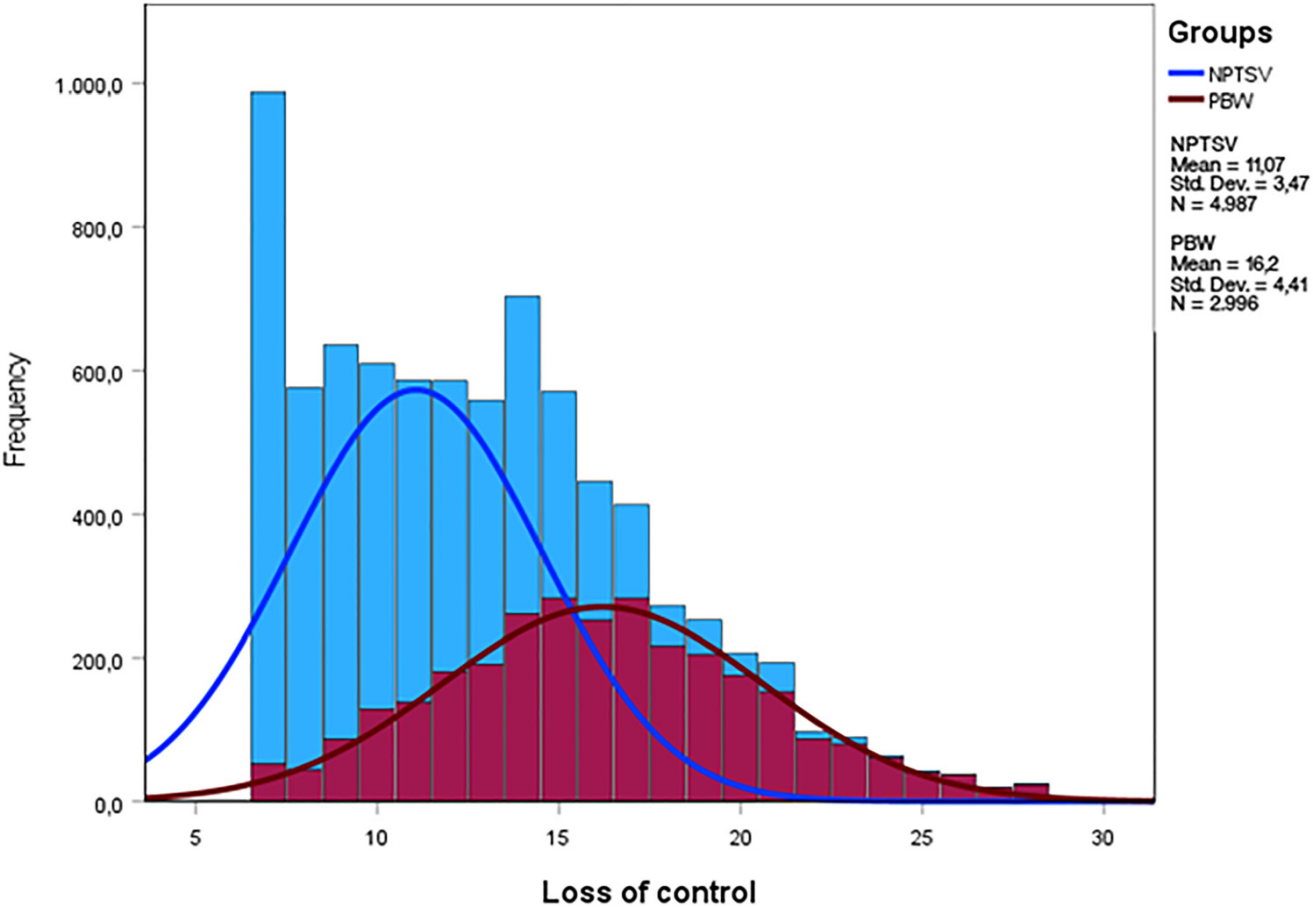
Frequency curves of scores of non-problematic TV series viewers and problematic binge-watchers for the loss of control facet of the BWESQ *Note.* BWESQ: Binge-Watching Engagement and Symptoms Questionnaire; PBW: problematic binge-watchers; NPTSV: non-problematic TV series viewers; Std. Dev.: standard deviation

In summary, the current results indicate that no reliable BWESQ cut-off scores could be determined to accurately discriminate problematic from non-problematic binge-watchers. They also point to the notion that applying such a diagnostic approach might not be the most relevant in the context of binge-watching behaviors. Notably, since most putative behavioral addictions (except gambling and gaming disorders) are not yet recognized as such in international diagnostic classifications, the current lack of established diagnostic criteria for problematic and potentially addictive engagement in these activities prevents the generation of reliable cut-off scores. This is why researchers and clinicians should, at this stage, refrain from proposing cut-off scores in new scales that assess emerging problematic behaviors, including the binge-watching research field as well as other emerging conditions. Indeed, previous attempts to suggest cut-offs for such scales (e.g., in the context of “Internet addiction”) resulted in unrealistic prevalence rates (up to 10%–20% of “pathological cases”; e.g., Kuss, Griffiths, Karila, & Billieux, 2014), thus promoting over-pathologization, stigmatization, and moral panic. Efforts should instead be focused on developing a strong research base to clarify where the dividing line between elevated but non-harmful and problematic patterns of engagement resides. Clinically useful assessment criteria could then be derived, thus allowing for the generation of valid cut-off scores in terms of measurement instruments specially designed for this purpose.

It is worth noting that determining reliable cut-off scores for self-reported screening tools (such as the BWESQ) requires a gold standard (e.g., a diagnostic interview administered by a certified clinician), which was not possible in the present context as binge-watching is not a recognized condition. We also want to point-out that the identification of problematic behaviors should go beyond the use of a single cut-off, and that different cut-offs could be used for different purposes. For example, we could opt for a different cut-off if our aim is to diminish the number of false positives to avoid over-pathologization effects, or if, in contrast, our objective is to reduce as far as possible false negatives to ensure that most persons in need of help are correctly identified via the screening instrument. Finally, future studies could also apply other statistical approaches (e.g., supervised machine learning) to identify optimal cut-off scores based on a selection of theoretically informed variables.

## Authors’ contribution

MF, JB, SB and PB designed the statistical analysis strategy. PB ran the statistical analyses. MF, JB, SB and PB interpreted the results. PB wrote the initial draft of the commentary under the supervision of MF and JB. MF, JB, SB and PM reviewed the initial draft and participated in the writing of the final draft. All authors approved the final version of the manuscript.

## Conflict of interest

PM is funded by the Belgian Fund for Scientific Research (FRS-FNRS, Belgium).

## Acknowledgments

The authors would like to warmly thank Robert Astur, Rafael Ballester-Arnal, Jesús Castro-Calvo, Gaëlle Challet-Bouju, Matthias Brand, Georgina Cárdenas, Gaëtan Devos, Hussien Elkholy, Marie Grall-Bronnec, Richard J.E. James, Martha Jiménez-Martínez, Yasser Khazaal, Saeideh Valizadeh-Haghi, Daniel L. King, Yueheng Liu, Christine Lochner, Sabine Steins-Loeber, Jiang Long, Marc N. Potenza, Shahabedin Rahmatizadeh, Adriano Schimmenti, Dan J. Stein, István Tóth-Király, Richard Tunney, Yingying Wang and Zu Wei Zhai for their precious support in collecting the data used in the present commentary.

## Footnotes

1 See Flayelle, Verbruggen, et al. (2020) for the rationale behind the selection of criteria used for creating the three groups.

## Appendix A

**Table A1.** Curve coordinates and associated sensitivity, specificity, positive and negative predictive values and accuracy of the binge-watching facet of the BWESQ

Value	Sensitivity	Specificity	PPV	NPV	Accuracy
5.000	1.000	0.000	0.375	/	0.375
6.500	0.996	0.091	0.397	0.974	0.431
7.500	0.992	0.153	0.413	0.970	0.468
8.500	0.985	0.234	0.436	0.962	0.516
9.500	0.972	0.313	0.459	0.948	0.560
10.500	0.946	0.394	0.484	0.924	0.601
11.500	0.903	0.484	0.513	0.892	0.641
12.500	0.845	0.605	0.563	0.867	0.695
13.500	0.770	0.706	0.611	0.836	0.730
14.500	0.675	0.799	0.669	0.804	0.752
15.500	0.561	0.863	0.712	0.766	0.750
16.500	0.433	0.914	0.751	0.729	0.733
17.500	0.318	0.953	0.803	0.699	0.715
18.500	0.223	0.973	0.832	0.676	0.691
19.500	0.159	0.984	0.856	0.661	0.674
20.500	0.109	0.992	0.891	0.650	0.661
21.500	0.072	0.996	0.911	0.641	0.649
22.500	0.044	0.998	0.942	0.635	0.640
23.500	0.021	0.999	0.955	0.630	0.632
25.000	0.000	1.000	/	0.375	0.375

*Note.* BWESQ: Binge-Watching Engagement and Symptoms Questionnaire; NPV: negative predictive value; PPV: positive predictive value.

## Appendix B

**Table B1.** Curve coordinates and associated sensitivity, specificity, positive and negative predictive values and accuracy of the loss of control facet of the BWESQ

Value	Sensitivity	Specificity	PPV	NPV	Accuracy
6.000	1.000	0.000	0.375	/	0.375
7.500	0.983	0.188	0.421	0.947	0.486
8.500	0.968	0.295	0.452	0.939	0.547
9.500	0.939	0.405	0.486	0.914	0.604
10.500	0.896	0.501	0.519	0.889	0.650
11.500	0.850	0.591	0.556	0.868	0.688
12.500	0.790	0.673	0.592	0.842	0.717
13.500	0.726	0.746	0.632	0.819	0.739
14.500	0.639	0.835	0.700	0.794	0.761
15.500	0.544	0.893	0.753	0.765	0.762
16.500	0.460	0.932	0.802	0.742	0.755
17.500	0.366	0.958	0.840	0.715	0.736
18.500	0.294	0.970	0.853	0.696	0.716
19.500	0.225	0.979	0.866	0.678	0.696
20.500	0.167	0.985	0.872	0.663	0.678
21.500	0.115	0.994	0.915	0.652	0.664
22.500	0.086	0.996	0.925	0.645	0.655
23.500	0.060	0.998	0.942	0.639	0.646
24.500	0.040	0.999	0.976	0.510	0.639
25.500	0.026	0.999	0.952	0.631	0.634
26.500	0.014	0.999	0.933	0.628	0.630
27.500	0.008	1.00	0.920	0.626	0.627
29.000	0.000	1.000	/	0.625	0.625

*Note*. BWESQ: Binge-Watching Engagement and Symptoms Questionnaire; NPV: negative predictive value; PPV: positive predictive value.
